# Hybrid Metalens–Compressive Sensing Imaging System for Ultra‐Low‐Power Edge Detection

**DOI:** 10.1002/nap2.70079

**Published:** 2026-04-16

**Authors:** Sejin Yoon, Hasung Kim, Hyunkeun Lee, Trevon Badloe

**Affiliations:** ^1^ Department of Electronics and Information Engineering Korea University Sejong South Korea; ^2^ Department of AI Semiconductor Engineering Korea University Sejong South Korea; ^3^ Division of Smart Energy Convergence Engineering Korea University Sejong South Korea; ^4^ Digital Healthcare Center Sejong Institute for Business and Technology, Korea University Sejong South Korea

**Keywords:** compressive sensing, edge‐enhanced imaging, metalens, opto‐electronic computing

## Abstract

Low‐power edge detection is essential for next‐generation vision systems that require real‐time on‐device processing of optical information. Conventional imagers rely on power‐intensive digital postprocessing for edge detection, creating a data bottleneck between the image sensor and signal processor. We propose a system‐level hybrid optoelectronic edge imaging framework that combines an analog optical computing metalens with a compressive sensing CMOS image sensor (CS‐CIS). The spiral metalens with a topological charge of l=+2 directly images and encodes second‐order edge information in the optical domain, eliminating the need for additional bulky optics and digital edge extraction algorithms. This optically processed information is captured by the CS‐CIS that selects a reduced number of pixel values, minimizing redundant data acquisition and enabling high‐speed and ultra‐low‐energy operation. Simulation results validate the effectiveness of this approach, demonstrating a peak signal‐to‐noise ratio of 23.87 dB at a 25.0% compression rate. This work paves the way for energy‐efficient edge‐aware systems with potential applications in robotics and computer vision.

## Graphical abstract

We propose an ultra‐low‐power edge detection imaging system through the combination of a spiral metalens and a compressive sensor. By moving the edge detection to a passive optical element, not only is the required power reduced but also the encoded edge image provides rich high‐frequency information, which provides high‐fidelity image reconstruction after compression.

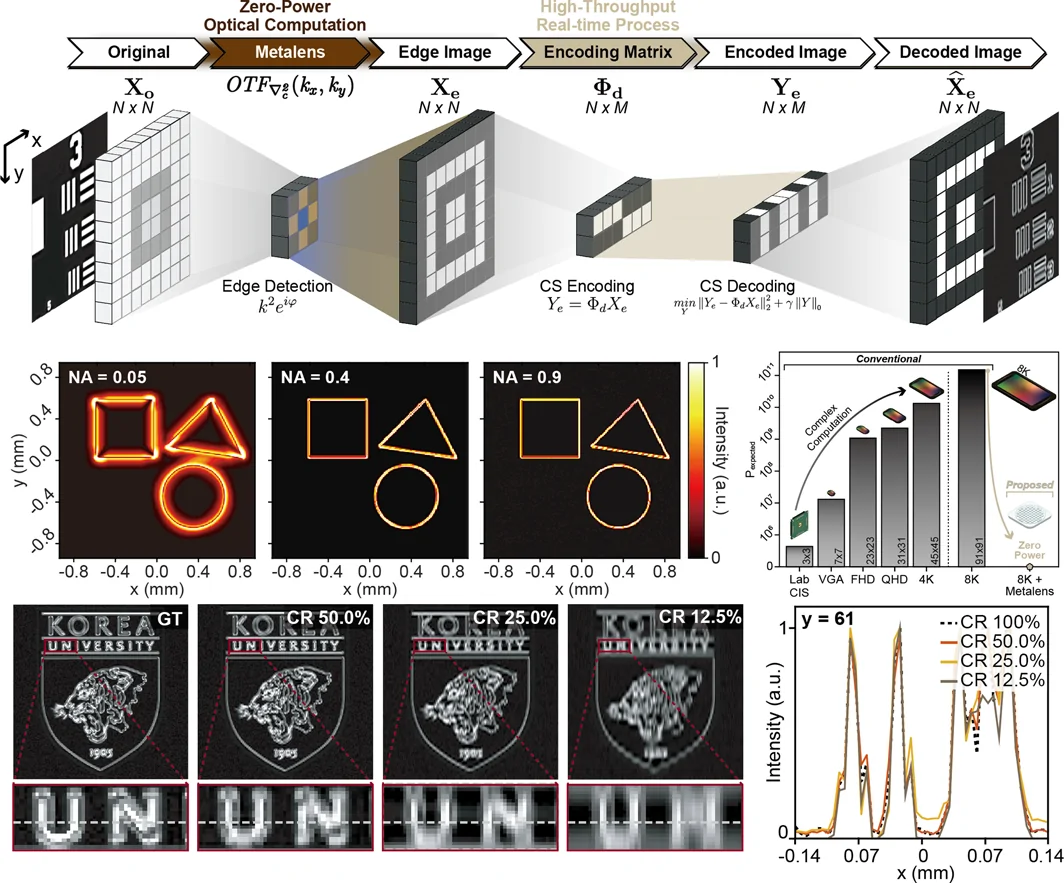

## 1 Introduction

The explosive growth of data‐driven technology has placed immense demand on real‐time image processing techniques. Edge detection, the process of identifying the boundaries of an image, is an important step in various machine vision pipelines, such as object detection in robotics and autonomous systems [1, 2, 3, 4]. Although conventional digital methods based on computational kernels and algorithms are typically compact and achieve high accuracy, the processing of high‐resolution data in real time is computationally expensive and results in significant latency [5, 6]. To address this, on‐chip edge detection processors have been implemented, thereby reducing external processing and the need for data transfer [7, 8, 9]. However, this still requires additional operational power and increases the circuit overhead, which can limit the functional pixel area and sensor performance [10]. Furthermore, they face inherent limitations where processing time and edge detection power consumption scale upward as resolution increases.

Recently, direct edge detection in the optical domain has been demonstrated as a promising alternative, allowing analog calculations without additional power consumption at the speed of light [11, 12]. Optical differentiators have been proven with multilayer structures [13], nonlocal metasurfaces [14], and through the spin Hall effect of light [15]. Metasurfaces, quasi‐2D flat optical devices consisting of subwavelength structures known as “meta‐atoms,” enable precise, multifunctional control of the phase and amplitude of light within a single element [16]. With the inclusion of a lens phase in a metasurface, metalenses with various functionalities have been utilized for complex mathematical operations with a compact footprint [17, 18]. However, optical systems still require full‐pixel sensor readout into the digital domain.

Compressive sensing (CS) provides a powerful synergy to address this limitation. By exploiting the sparsity of image signals in the transformed domain, CS enables the reconstruction of images with less data than required to satisfy the Shannon–Nyquist theorem, thereby reducing acquisition time and power [19]. The principles of CS have also been embedded directly into CIS, enabling the realization of computational imaging [20, 21]. To implement an integrated edge‐detecting compressive sensing CMOS image sensor (CS‐CIS), the edge‐detecting operator must be chosen considering the CS reconstruction fidelity. First‐derivative operators (e.g., Sobel and Prewitt) are simple to implement but discard substantial low‐frequency content, whereas second‐derivative operators (e.g., the Laplacian) retain more information but are difficult to implement on‐sensor [22, 23].

Although spiral phase filtering and compressive sensing have each been extensively studied separately, here, we demonstrate a system‐level optoelectronic codesign that couples optical edge encoding with on‐chip compressive acquisition. Importantly, when combining CS with edge detection, the placement of the edge operator is a critical design choice because performing edge extraction before or after compression leads to fundamentally different reconstruction fidelity and hardware requirements. To address this, we conduct a quantitative study on the operation ordering and show that an optical preprocessing stage prior to CS acquisition is advantageous. We also conduct system‐level optical simulations under practical sensor sampling constraints to quantify the trade‐offs between metalens numerical aperture and edge imaging performance, thereby guiding hardware‐oriented metalens design. Building on these rationales, we propose a compact, ultra‐low‐power, high‐speed optoelectronic imaging framework for edge detection.

Figure 1a compares our proposed hybrid imaging system with conventional computational edge detection methods. Although conventional pipelines require computation on full‐frame sensor data, we shift the second‐order edge imaging function into the optical domain through the use of a spiral metalens. This reduces the required computational power and time for edge detection to zero and to the speed of light, respectively. As the optical computation is an analog process, the entire image is processed at once, regardless of the resolution. To further decrease the sensor readout requirements, we employ a CS‐CIS for data compression. A comparison between our proposed system and other on‐chip electronic edge detection and optical edge detection methods is presented in Table 1, highlighting the benefits of our optoelectronic system in terms of high frame rate operation and low power consumption. Figure 1b shows the reconstructed edge image obtained using only 25% of the original data, demonstrating edge image quality comparable to that of a conventional computational system. Our simulations show that the proposed framework maintains accurate edge information even at high compression rates (CR of 25%) with an average reconstruction accuracy of 23.87 dB across 10 distinct images.

**FIGURE 1:**
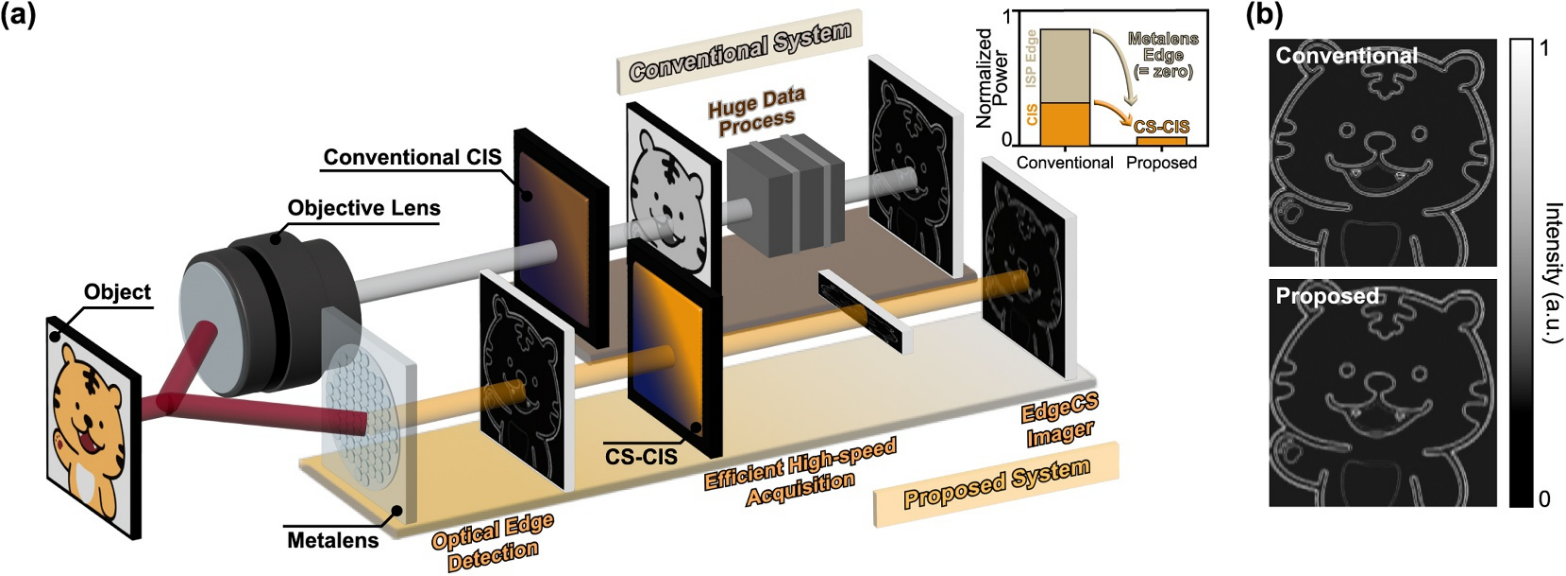
Schematic illustration of the hybrid optoelectronic edge imaging system. (a) Comparison between the conventional and proposed hybrid edge detection systems. (b) Edge detection results using the conventional and proposed systems.

**TABLE 1:** Detailed comparison with recent relevant work.

		[17]	[24]	[7]	[9]	This work	
Method		Optical (metalens)	Optical (Fourier filter)	Electronic (on‐chip)	Electronic (on‐chip)	Optical (metalens) + electronic (on‐chip compressive sensing)	
Pixel resolution		1616 × 1216	1280 × 1024	192 × 128	1296 × 976	256 × 256	
Frame rate [fps] ↑		12	30	312.5	1000	781 (@CR = 100.0%)	6248 (@CR = 12.5%)
Total power consumption [mW] ↓	Sensor	2500	1100	49.2	331	56.38	56.38
	Edge detection	0	0	3.3	32	0	
^a^FoM_1_ [nJ/pixel/frame] ↓	Original	0	0	0.43	0.03	0	0
	4K	0	0	145.02	0.17	0	0
	8K	0	0	580.08	0.66	0	0
^b^FoM_2_ [nJ/pixel/frame] ↓		106.02	27.97	6.84	0.29	1.10	0.14

^a^ FoM_1_ **=** (edge detection power consumption)/(pixel resolution × frame rate).

^b^ FoM_2_ = (total power consumption)/(pixel resolution × frame rate).

## 2 Results and Discussion

### 2.1 Edge Detection Mechanisms

Most classic edge detection operators (e.g., Sobel and Prewitt) are first‐order derivative operators. These operators identify edges by calculating the magnitude of the image gradient, determining the point at which the intensity change between adjacent pixels is steepest. This image gradient is fundamentally a 2D vector that quantifies the rate and direction of intensity change. The standard operator for approximating the gradient (∇) in an image is the Sobel operator. In a 2D Cartesian coordinate system (*x*, *y*), this gradient is defined by its partial‐derivative components:

(1) $$\begin{array}{c} \nabla =\left[\frac{\partial }{\partial x},\frac{\partial }{\partial y}\right]=\left(d_{x},d_{y}\right). \end{array}$$

The final edge map is then computed as the magnitude of this gradient vector, resulting in an effective first‐order differentiation operation:

(2) $$\begin{array}{c} G\left(x,y\right)=\left\|\nabla \right\|=\sqrt{{d_{x}}^{2}+{d_{y}}^{2}}=\sqrt{{\frac{\partial }{\partial x}}^{2}+{\frac{\partial }{\partial y}}^{2}}. \end{array}$$

However, this approach has significant limitations. The two separate convolution operations result in a high computational cost, and as can be seen in the first‐derivative results of Figure 2a, the maximum value of the gradient can extend over a wide area. This produces thick edges, limiting the precision of locating specific edge features.

**FIGURE 2:**
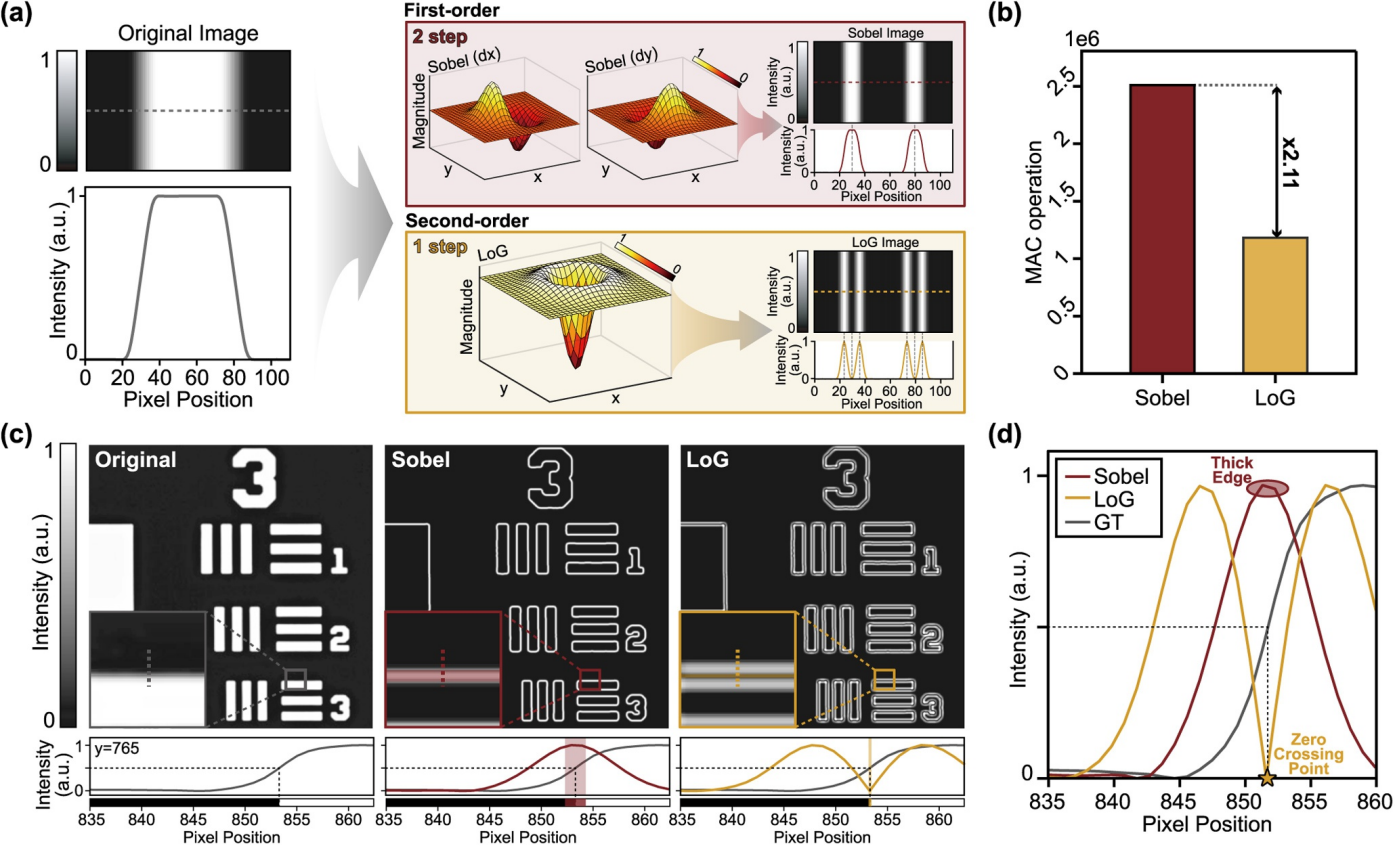
Principle of edge detection with edge kernels. (a) Schematic of edge detection using Sobel and LoG operations. (b) Comparison of the number of MAC operations among the operators. (c) Results of theoretical calculations and zoomed‐in region (inset). (d) Comparison of the cross‐sectional intensity profiles along the dashed lines indicated in the inset of (c).

On the other hand, the second‐order derivative calculates the rate of change of the image gradient, allowing edge detection with a zero‐crossing point that appears at a specific location, allowing precise edge information. In addition, second‐order derivative operators tend to detect object boundaries as continuous, closed contours, which is very useful for image segmentation [19]. The standard operator for isotropic second‐order differentiation is the Laplacian (∇^2^). In a 2D Cartesian coordinate system (*x*, *y*), this is defined as follows:

(3) $$\begin{array}{c} \nabla^{2}=\frac{\partial^{2}}{\partial x^{2}}+\frac{\partial^{2}}{\partial y^{2}}. \end{array}$$

To create a band‐limited and robust version of this operator, it is commonly convolved with a Gaussian kernel [25, 26], G(x,y;σ), resulting in the Laplacian of Gaussian (LoG) filter:

(4) $$\begin{array}{c} \mathrm{LoG}\left(x,y\right)=-\frac{1}{\pi \sigma^{4}}\left[1-\frac{x^{2}+y^{2}}{2\sigma^{2}}\right]e^{-\frac{x^{2}+y^{2}}{2\sigma^{2}}}. \end{array}$$

As illustrated in Figure 2a, the LoG operator creates a zero‐crossing point with a single‐pixel width, enabling highly precise edge localization.

A comparison of the computational load for each operator is shown in Figure 2b, quantified by the number of multiply‐accumulate (MAC) operations. As the Sobel kernel requires two convolution operations, it requires a computational load that is over 2 × that of the LoG. To see the difference between the two edge detection operators, we use the 1951 United States Air Force (USAF) resolution test chart as the target and apply each operator computationally (Figure 2c). The first‐order derivative Sobel kernel results in a thick peak at the image boundary, whereas the second‐order derivative LoG kernel detects a precise edge through the zero‐point crossing. This is displayed through 1D cross‐sections of the images in Figure 2d, comparing the original, Sobel and LoG profiles. Because the Sobel kernel forms a thick, single edge, it is difficult to accurately discriminate the true edge during the subsequent thresholding process. In contrast, the LoG kernel generates a precise zero‐crossing point at a specific location, substantiating the accuracy of the operator. Therefore, we choose a second‐order derivative as the focus of this work while additionally reducing the number of MAC operations of this function to zero through an analog optical implementation.

### 2.2 Compressive Sensing Imaging Framework

A conventional CIS acquires a full‐resolution frame by sequentially selecting each row/pixel and reading out every pixel value. This results in high readout bandwidth and energy consumption due to per‐pixel conversion and data transfer. In contrast, a CS‐based CIS directly reads out a reduced set of compressed measurements, so fewer operations and less time are required for each frame [20]. Here, we adopt a CS‐CIS sampling network proposed in previous work [21]. In this method, for each column, vertically contiguous pixels are grouped into blocks and oversampled across acquisition cycles, then combined using a predefined weighting sequence and averaged to produce compressed linear measurements in a block‐by‐block manner. This sensing process can be modeled as a standard compressive sensing equation. An image, *X* (with N × N pixels), with a sparse representation s via the basis ψ compressed by using a measurement matrix Φ (M × N) is given as follows:

(5) $$\begin{array}{c} Y=\Phi X=\Phi \psi s. \end{array}$$

For example, an original 256 × 256‐pixel image can be compressed directly by the sensor to 64 × 256 measurements, achieving a 4 × compression rate (CR = 25%) at the time of acquisition.

To reconstruct the original image from the compressed measurements, we employ the adaptively learned sparsifying basis (ALSB) algorithm. ALSB adaptively learns a patch‐optimized sparsifying basis, enabling an efficient solution of the reconstruction problem and offering the practical advantage that it does not require a pretrained model. It is well suited to edge images because edges are locally sparse and nonstationary, and the ALSB overlapped‐patch sparse model preserves sharp transitions while reducing blocking artifacts [27]. The reconstruction process of this algorithm is a mathematical optimization problem that finds the sparse signal $\hat{s}$ using the compressed measurements, the measurement matrix Φ, and a sparsifying matrix ψ and finally restores the image through the operation as follows:

(6) $$\begin{array}{c} \min_{s}{\left\|Y-\Phi \psi s\right\|}_{2}^{2}+\gamma {\left\|s\right\|}_{0}. \end{array}$$

The sparsifying transform matrix is optimized by learning the unique characteristics of the image directly from the input data, thereby maximizing reconstruction quality.

A comparison between the operational sequence of the conventional edge detection system and our proposed framework is shown in Figure 3a. Conventionally, after the image is acquired and reconstructed via compressive sensing, edge detection must be performed digitally. However, as shown in Figure 3b, as images generally consist mainly of low‐frequency components, the high‐frequency components are lost during reconstruction, leading to blurred edges. This is particularly severe at high CR, as the image boundaries are significantly degraded, resulting in a low‐quality edge image. By inverting this process, the image being compressed is already rich in high‐frequency components that constitute the edge information. We evaluate our system’s robustness to noise in Figure 3c,d. We apply an additive white Gaussian noise model that approximates sensor noise. The LoG‐filtered result of the noise‐free original image is used as the ground truth. We then generate noisy inputs by adding noise to the original image with root mean square intensity levels of 0.5%, 1%, 2%, 3%, 5%, and 10%. After applying the LoG kernel to each noisy image, we perform compressive sensing acquisition and reconstruction and compare the reconstructed results with the ground truth. The results show that, across all compression ratios, the reconstruction accuracy—evaluated using PSNR and structural similarity index measure (SSIM)—remains comparable down to a noise intensity of approximately 5%. This indicates that, when considering sensor noise, the optical preprocessing using the Gaussian characteristics of the LoG kernel provides noise attenuation benefits, leading to robust reconstruction accuracy in compressive sensing imaging. Then, we measure the performance of this inverted framework at various CRs across 10 images and quantify them through the peak signal‐to‐noise ratio (PSNR). Using the conventional method of computationally applying a LoG filter to a reconstructed image as the benchmark, we find that our method of preprocessing the image before reconstruction provides higher edge reconstruction accuracy (Figure 3e **).** This is especially evident at higher compression rates throughout the entire dataset (Figure 3f). Although this maximizes the theoretical advantages of CS technology, providing a higher‐fidelity edge image after reconstruction, the edge detection kernel must still be computationally performed on the full‐resolution image. This energy‐intensive operation can be replaced with passive optical devices that have been proven for power‐free, speed‐of‐light, analog edge‐enhanced imaging [28, 29, 30].

**FIGURE 3:**
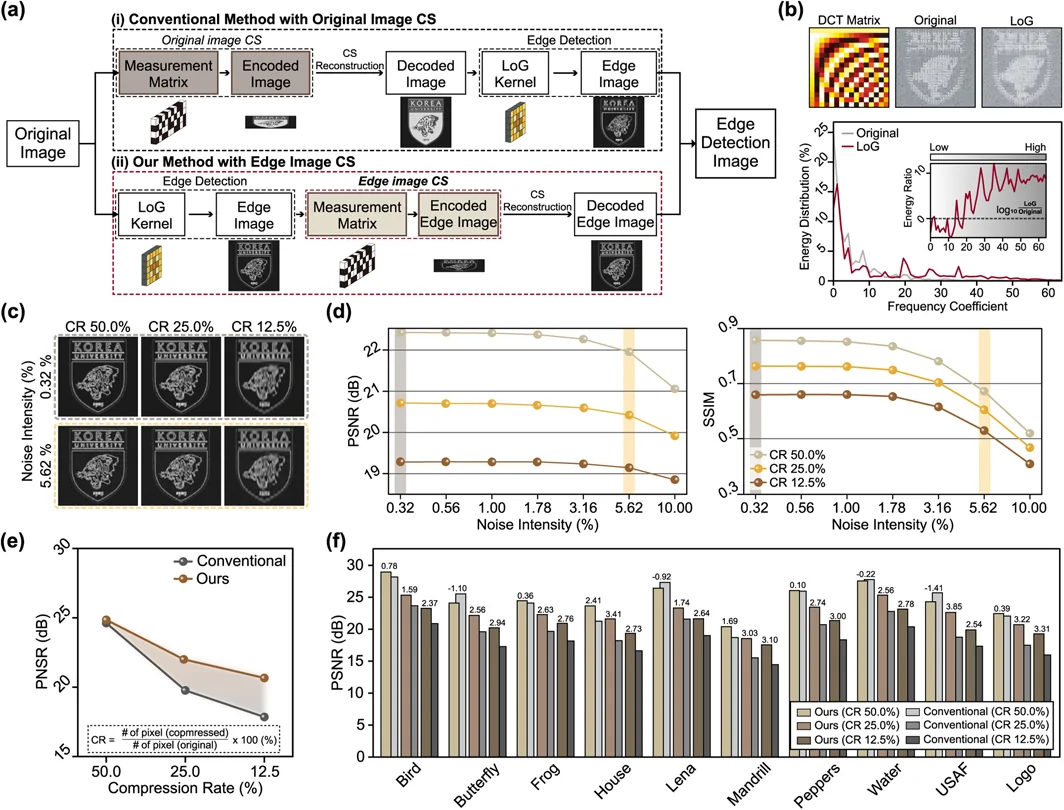
Comparison of compressive sensing imaging pipelines. (a) Block diagram showing the sequence of the edge detection imager using the conventional system and our system. (b) Sparsity in the discrete cosine transform (DCT) domain according to the original image and LoG‐filtered image, showing the energy distribution per frequency coefficient and their ratio (inset). (c) Visual evaluation of reconstruction results under various noise intensities and (d) reconstruction performance (PSNR and SSIM) versus noise intensity for each compression ratio. (e) Average PSNR performance versus CR for the entire dataset and (f) individual images. The numbers above each bar represent the PSNR difference between the two methods.

### 2.3 Optical Implementation of Second‐Order Differentiation

#### 2.3.1 LoG Kernel

We find the corresponding optical transfer function (OTF) of the LoG kernel by taking its Fourier transform. In the spatial frequency domain $k_{x},k_{y}$,

(7) $$\begin{array}{c} H\left(k_{x},k_{y}\right)=F\left\{\mathrm{LoG}\left(x,y\right)\right\}=-\left(k_{x}^{2}+k_{y}^{2}\right)e^{-{2\pi^{2}\sigma }^{2}\left(k_{x}^{2}+k_{y}^{2}\right)}. \end{array}$$

By placing a physical mask or filter with this OTF into the Fourier plane of a 4f optical imaging system, analog optical filtering of the incident wavefront can be performed at the speed of light in the frequency domain (Figure 4a). We create a target image made up of a square, circle, and triangle to highlight how curves and edges at all angles and directions are processed by the isotropic kernel, determined by the $k_{x}^{2}+k_{y}^{2}$ term in Equation (7) (Figure 4b). Processing this image with the LoG kernel shows that the edge detection takes place in the real part of the electric field, with an isotropic double‐edge feature and a zero‐crossing point in the center.

**FIGURE 4:**
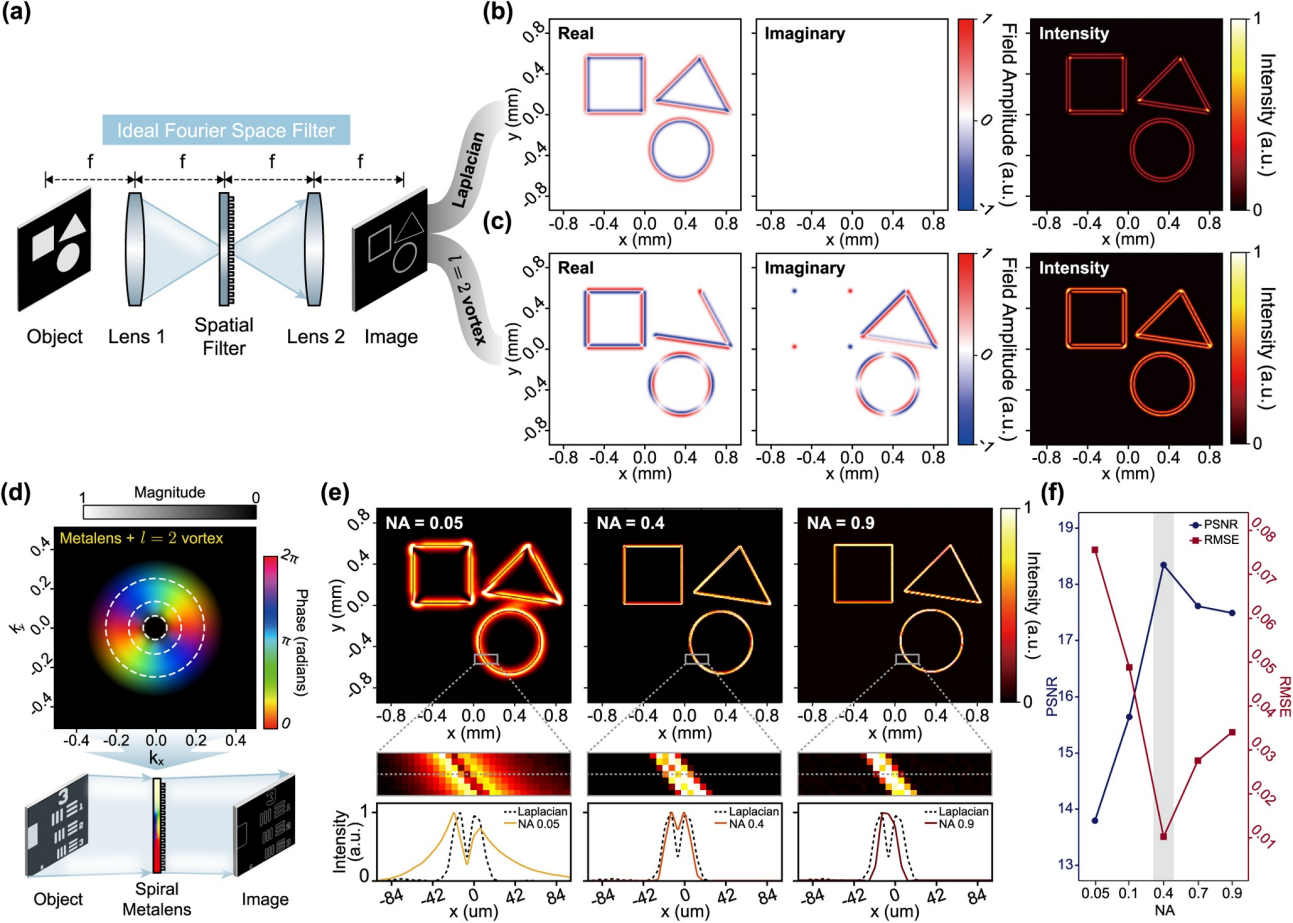
Optical implementation of second‐order differentiation. (a) Schematic of a 4f imaging setup with a spatial filter for analog edge image processing. The real and imaginary parts of the electric field, and the resulting detected intensity after being processed with (b) a Laplacian filter and (c) an l=+2 vortex filter. (d) Schematic of the edge imaging setup using a spiral metalens with l=+2 and the required complex PSF. (e) The output edge images through the spiral metalens with an NA of 0.05, 0.4, and 0.9, with zoomed‐in sections of the triangle and plots of their 1D cross‐sectional intensity. (f) PSNR comparison of the simulated images and RMSE analysis of the 1D cross‐sectional intensity plots for different NA values.

However, the LoG kernel is intrinsically bipolar, with a positive central lobe and a surrounding negative ring (Figure 2a), which makes it impossible to implement in a single passive optical element, as optical intensity must be positive. Workarounds have been demonstrated using hybrid optical‐electronic approaches, such as encoding the positive and negative components into orthogonal channels through polarization multiplexing [31]. The final, true LoG‐filtered image must then be reconstructed by the electronic subtraction of the two captured channels. As this requires additional computational power and optical components to control the polarization of light, here, we investigate an alternative approach to achieve second‐order differentiation, namely, using a vortex phase with a topological charge of l=+2.

#### 2.3.2 Second‐Order Vortex

The justification for choosing a spiral metalens with a topological charge of l=+2 is rooted in the specific requirements of high‐precision second‐order edge detection. A topological charge of l=+1 performs first‐order spatial differentiation, which, similar to conventional gradient‐based operators, typically produces thicker edges that limit edge localization [32]. In contrast, employing higher‐order topological charges enlarges the central phase singularity of the point spread function. This expansion causes a suppression of low‐spatial‐frequency components, leading to a severe loss of spatial information [33]. Consequently, l=+2 provides the optimal balance, enabling accurate and omnidirectional second‐order edge extraction without sacrificing essential spatial details.

To achieve this, we start with a continuous analog of the complex Sobel filter $H_{1}=H_{x}+iH_{y}:$

(8) $$\begin{array}{c} \nabla_{c}=\frac{\partial }{\partial x}+i\frac{\partial }{\partial y}. \end{array}$$

The OTF required to implement this operator is linear in spatial frequency:

(9) $$\begin{array}{c} \text{OT}F_{\nabla c}\left(k_{x},k_{y}\right)\propto k_{x}+ik_{y}=ke^{i\varphi }. \end{array}$$

Here, *k* is the radial spatial frequency and φ is the azimuthal angle. This pupil function is the definition of a spiral phase plate with a topological charge of l=+1.

By multiplying this function in the Fourier domain, we can achieve the second‐order operator to construct the second‐order operator $\nabla_{c}^{2}$:

(10) $$\begin{array}{c} \text{OT}F_{\nabla c^{2}}\left(k_{x},k_{\gamma }\right)\propto {\left(k_{x}+ik_{y}\right)}^{2}=k^{2}e^{i2\varphi }. \end{array}$$

This pupil function can be physically realized by a metasurface imparting a spiral phase with a topological charge of l=+2. To understand its behavior in the spatial domain, we can expand the squared operator as follows:

(11) $$\begin{array}{c} \nabla_{c}^{2}={\left(\frac{\partial }{\partial x}+i\frac{\partial }{\partial y}\right)}^{2}=\left(\frac{\partial^{2}}{\partial x^{2}}-\frac{\partial^{2}}{\partial y^{2}}\right)+2i\frac{\partial^{2}}{\partial x\partial y}. \end{array}$$

Compared to Equation (3), a subtraction between the ∂^2^/∂*x* ^2^ and ∂^2^/∂*y* ^2^ terms appears, which breaks the rotational symmetry, creating an anisotropic OTF. The real part of the operator, ($\frac{\partial^{2}}{\partial x^{2}}-\frac{\partial^{2}}{\partial y^{2}}$), is maximally sensitive to horizontal and vertical edges, whereas it cancels to 0 for edges oriented at ±45°. Conversely, the imaginary part, $2i\frac{\partial^{2}}{\partial x\partial y}$, is maximally sensitive to diagonal edges and 0 for horizontal and vertical ones. These features are clearly seen in the real, imaginary, and intensity images of the target image processed with the l=+2 vortex in Figure 4c. It is evident that the location of the positive and negative field amplitude flips is in accordance with the rotation of the phase defined by φ [34]. The final intensity image captured on a CCD, $I={\left|\text{Object}\times \text{PS}F_{\nabla c^{2}}\right|}^{2}$, combines the energy from both the real and imaginary channels, providing second‐order differentiation of edges of all orientations as desired. Although the intensity of the detected edge is modulated by its orientation, which can lead to bright spots at corners and regions of high curvature, the output intensity of the image is comparable with that of the LoG operation, with no attenuation at edges at ± 45°. Most importantly, as the operator required for the vortex is complex‐valued rather than bipolar, its PSF can be generated with a single phase mask, allowing the physical realization using a single static metasurface, without any additional computational requirements.

#### 2.3.3 Spiral Metalens

Although metasurfaces can be used as Fourier domain filters, their multifunctionality also allows a lens phase to be integrated directly into the OTF. By combining a hyperbolic lens phase ($\phi_{\text{lens}}$) with the required spiral phase profile, a spiral metalens can be designed [35, 36, 37]. This allows for a dramatic reduction in the size of the overall system, down to a single analog imaging processing lens and CS‐CIS. The numerical aperture (NA) of a lens measures how much light can be gathered and is defined as follows:

(12) $$\begin{array}{c} \text{NA}=n\;\sin\left(\theta \right) \end{array},$$

where θ is the half‐angle of the maximum cone of light that can enter the lens. Figure 4d shows the required complex PSF for a spiral metalens created with a hyperbolic lens phase and a topological charge of l=+2. The double spiral is evident in that each color is represented twice, whereas the donut‐shaped magnitude is represented by the saturation of the color. Three dashed circles are highlighted, representing low‐, medium‐, and high‐NA lenses. It is evident that a high‐NA metalens is able to collect light from larger angles, that is, high values of $k_{x}\;\text{and}\;k_{y}$, which relate to high spatial frequencies, as well as resolve finer diffraction‐limited details. We investigate how this affects the optical image processing directly through the metalens.

Metalenses with various NAs are simulated using the angular spectrum method, and their PSFs are used as the kernel for a convolution with the same target image from Figure 4a. The results are shown in Figure 4e for NA values of 0.9, 0.4, and 0.05, showing the difference between high‐, medium‐, and low‐NA lenses with a sensor size of 256 × 256 pixels of 6.5 μm pitch.

The high‐NA metalens produces extremely sharp features with high resolution, whereas the lower‐NA lenses produce a much more blurred edge image; however, they still maintain the zero‐crossing feature of the second‐order operation. Compared to the results of the spiral phase plate (Figure 4c), the inclusion of a focusing lens phase means that azimuthal components of the output field exist alongside the radial components from the vortex phase, resulting in edges in all directions being computed in both the real and imaginary parts of the output field. As the NA increases, the zero‐crossing feature is lost due to being finer than the maximum resolution of the simulated image sensor (Figure 4e). For quantitative validation, Figure 4f reports the PSNR of the simulated edge images (with respect to the LoG‐filtered original image as the reference) for different NA values, together with the RMSE of 1D cross‐sectional intensity profiles extracted at the same representative location. These results show that NA = 0.4 yields the closest match to the desired LoG edge output. Therefore, we use a spiral metalens with an NA of 0.4 for the rest of the investigation.

### 2.4 Hybrid Optoelectronic Edge Imaging System

A hybrid optoelectronic architecture that combines an analog optical computing metalens with an energy‐efficient CS‐CIS maximizes the advantages of each technology to create a compact, ultra‐low‐power solution for edge imaging. As illustrated in Figure 5a, the framework begins with the optical computation of an N × N grayscale image into an N × N edge image by the spiral metalens. Subsequently, the spatial encoding by the CS‐CIS compresses this edge data using an N × M encoding matrix, resulting in an N × M compressed edge image. The reconstruction algorithm then processes the compressed data to successfully reconstruct the edge image to its original N × N size. Three examples of images reconstructed using our spiral metalens and CS‐CIS system at different levels of compression are shown in Figure 5b. Compared to the reference image, the reconstructed images with CR = 50% and CR = 25% are visibly comparable, even when zoomed in to small features, whereas the CR = 12.5% image shows a slight deviation for smaller features. The average reconstruction performance, quantified by the PSNR, across the entire dataset of 10 images is shown in Figure 5c, by comparing the reconstructed results against the l=+2 vortex edge output of the original image as an ideal reference. This demonstrates that, by combining optical preprocessing with effectively zero computational power and CS‐based sampling to alleviate bandwidth limitations, our system can still achieve comparable performance at CR = 25.0%. To demonstrate the speed of image capture, we investigate the frame rate at various CRs (Figure 5d). As the CR increases, the achievable frame rate increases dramatically compared with the time required to acquire one full, uncompressed (CR = 100%) image. Although the performance at high compression (CR = 12.5%) shows limited performance (Figure 5b), CR = 25% provides a balance between quality and high frame rate. The image size and resolution are also important factors for edge detection. For conventional ISP‐based filtering, the computational load increases significantly with resolution, as larger images require the processing of more pixels and often larger kernel sizes. In contrast, in our system, the edge detection is performed optically by a passive optical element, requiring zero processing power. In Figure 5e, we compare the required power for edge detection for different sensor resolutions. This power requirement is calculated by multiplying the total sensor resolution (horizontal × vertical pixels) by the K × K kernel size.

**FIGURE 5:**
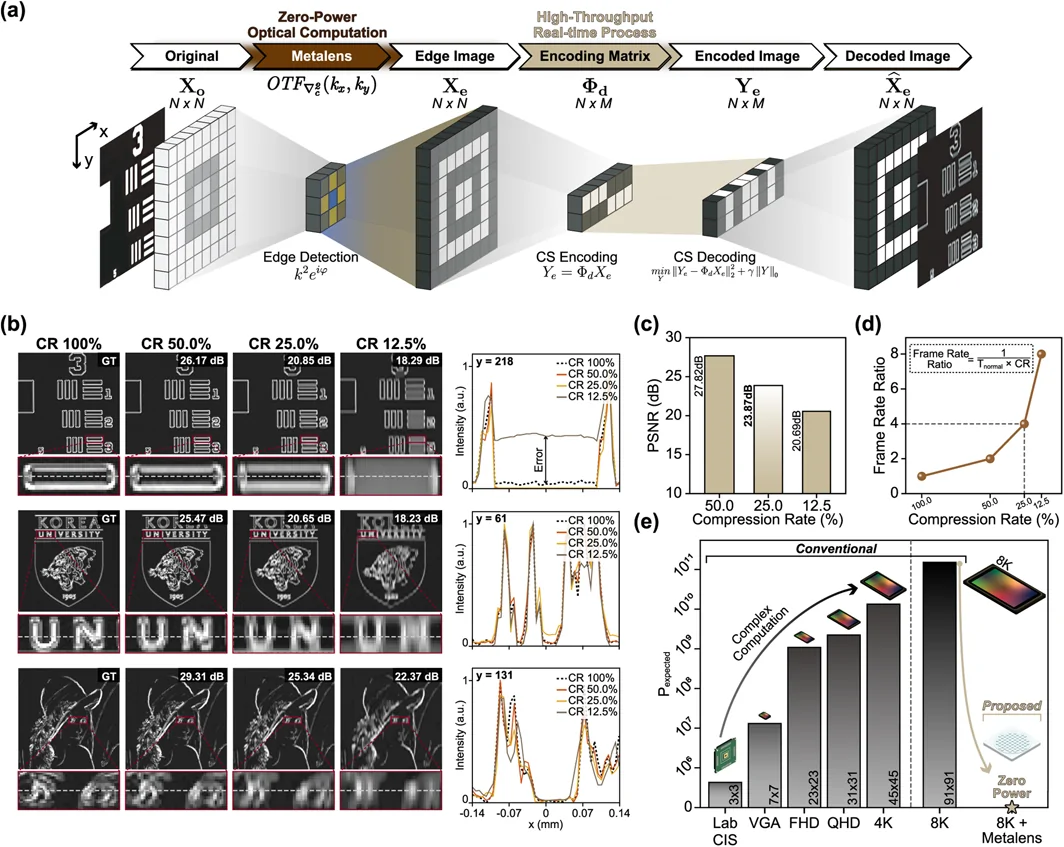
Simulated spiral metalens CS‐CIS imaging results. (a) Schematic diagram of the proposed system design. (b) Reconstructed images and cross‐sectional plots for three images (USAF, Symbol, Lena). The numbers in the top‐right corner of the image indicate the PSNR. (c) Comparison of the reconstruction performance for the entire dataset at various CRs. (d) Comparison of the frame rate at various CRs. (e) Comparison of the expected power for edge detection by sensor resolution.

## 3 Conclusion

We have proposed an ultra‐low‐power, high‐fidelity edge‐enhanced imaging system through the combination of a spiral metalens and CS‐CIS and demonstrated its feasibility through extensive simulations. The metalens acts as a front‐end encoder that takes the input image and provides an edge‐enhanced image directly to the sensor through an l=+2 topological charge vortex. Although compared to a standard isotropic LoG filter, the vortex provides an anisotropic edge‐detected image, the same double‐edge feature with a zero‐crossing is created and allows for reconstruction of the edge image through the CS‐CIS system even at high compression rates. Compared to conventional computational edge detection, our system demonstrates zero power consumption for realizing edge detection in the optical domain and an average reconstruction PSNR = 23.87 dB at a CR of 25%, achieving high‐speed operation with the CS‐CIS in the electronic domain. Modern AI vision systems increasingly face a system‐level bottleneck in moving massive raw data through the memory hierarchy and across high‐bandwidth memory interfaces [38, 39, 40]. As input resolution scales, the energy overhead of data transfer can dominate the required system power. By reducing the data volume generated at the source, the proposed system alleviates this transfer bottleneck and improves power efficiency for next‐generation AI‐driven vision systems. This framework could unlock advancements in on‐device remote imaging systems that require low power consumption and high throughput, such as in computer vision, robotics, and self‐driving vehicles.

Although, in this study, the output field is simulated through numerical calculations that require substantial computational power, moving these calculations to the optical domain using metalenses offers an additional advantage of being diffraction limited. The NA, and therefore the resolution, of the metalens can be chosen to match the required imaging performance and sensor specifications. Fabrication of such metalenses has been demonstrated with various nanofabrication techniques, from electron‐beam lithography to nanoimprint lithography, and could be implemented for this system [41]. To physically implement the desired phase map, isotropic meta‐atoms that rely on the propagation phase could be chosen for polarization‐independent operation and to reduce the footprint required for additional polarizers when using geometric phase. The precise fabrication of the designed meta‐atoms is paramount for peak performance of our proposed system, whereas discrepancies between the designed and fabricated metalens could result in reduced optical efficiency and additional aberrations that would reduce image fidelity [42]. As a potential solution for this, end‐to‐end optimization techniques using deep learning have been demonstrated, which would synergize seamlessly with the reconstruction algorithms required for CS [43, 44]. The physical realization of this system would also require the precise 3‐axis alignment of the metalens and the CS‐CIS to ensure accurate image formation directly on the sensor and potentially precalibration for the highest‐performance image reconstruction.

Beyond the operation demonstrated here, metalenses and metasurfaces are rapidly advancing toward reconfigurable and multifunctional platforms [45, 46, 47]. In particular, integrating active components such as liquid crystals and phase‐change materials into metasurfaces can provide a method of dynamic reconfigurability without additional external optics, providing a clear route to extend our metalens front‐end encoder toward an adaptive multimodal system. We envision future research exploiting the anisotropic nature of the l=+2 topological charge vortex with multiplexing metalenses to open new avenues of information detection, such as local orientation and chiral features, which would be extremely impactful for defect inspection and texture analysis.

## Author Contributions

**Sejin Yoon:** writing – original draft, investigation, data curation, formal analysis, validation, visualization. **Hasung Kim:** data curation, visualization. **Hyunkeun Lee:** conceptualization, methodology, resources, supervision, writing – review and editing, funding acquisition, project administration. **Trevon Badloe:** conceptualization, methodology, resources, supervision, writing – review and editing, funding acquisition, project administration.

## Funding

This work was supported by a Korea University grant (No. K2515531); a Korea Institute for Advancement of Technology (KIAT) grant funded by MOTIE, Korea (RS‐2025‐02215617, HRD Program for Industrial Innovation, 10%); the Alchemist Project grant funded by the Korea Planning & Evaluation Institute of Industrial Technology (KEIT) and the Korea Government (MOTIE) (Project Number: 1415185027‐20019169, 40%); and the Regional Innovation System & Education (RISE) program through the Sejong RISE Center, funded by the Ministry of Education (MOE) and the Sejong City, Republic of Korea (2025‐RISE‐08‐001). This work was partly supported by the Institute of Information & Communications Technology Planning & Evaluation (IITP)‐ITRC (Information Technology Research Center) grant funded by the Korea government (MSIT) (IITP‐2026‐RS‐2026‐25520273, 50%). [Correction added on 14 May 2026, after first online publication: The funding statement has been updated.]

## Conflicts of Interest

The authors declare no conflicts of interest.

## Data Availability

Data are available upon reasonable request.
